# Analysis of Heat Exposure During Pregnancy and Severe Maternal Morbidity

**DOI:** 10.1001/jamanetworkopen.2023.32780

**Published:** 2023-09-07

**Authors:** Anqi Jiao, Yi Sun, Chantal Avila, Vicki Chiu, Jeff Slezak, David A. Sacks, John T. Abatzoglou, John Molitor, Jiu-Chiuan Chen, Tarik Benmarhnia, Darios Getahun, Jun Wu

**Affiliations:** 1 Department of Environmental and Occupational Health, Program in Public Health, University of California, Irvine; 2 Institute of Medical Information, Chinese Academy of Medical Sciences and Peking Union Medical College, Beijing, China; 3 Department of Research & Evaluation, Kaiser Permanente Southern California, Pasadena; 4 Department of Obstetrics and Gynecology, University of Southern California, Keck School of Medicine, Los Angeles; 5 School of Engineering, University of California, Merced; 6 College of Public Health and Human Sciences, Oregon State University, Corvallis; 7 Department of Population and Public Health Sciences, University of Southern California, Los Angeles; 8 Scripps Institution of Oceanography, University of California, San Diego; 9 Department of Health Systems Science, Kaiser Permanente Bernard J. Tyson School of Medicine, Pasadena, California

## Abstract

**Importance:**

The rate of severe maternal morbidity (SMM) is continuously increasing in the US. Evidence regarding the associations of climate-related exposure, such as environmental heat, with SMM is lacking.

**Objective:**

To examine associations between long- and short-term maternal heat exposure and SMM.

**Design, Setting, and Participants:**

This retrospective population-based epidemiological cohort study took place at a large integrated health care organization, Kaiser Permanente Southern California, between January 1, 2008, and December 31, 2018. Data were analyzed from February to April 2023. Singleton pregnancies with data on SMM diagnosis status were included.

**Exposures:**

Moderate, high, and extreme heat days, defined as daily maximum temperatures exceeding the 75th, 90th, and 95th percentiles of the time series data from May through September 2007 to 2018 in Southern California, respectively. Long-term exposures were measured by the proportions of different heat days during pregnancy and by trimester. Short-term exposures were represented by binary variables of heatwaves with 9 different definitions (combining percentile thresholds with 3 durations; ie, ≥2, ≥3, and ≥4 consecutive days) during the last gestational week.

**Main Outcomes and Measures:**

The primary outcome was SMM during delivery hospitalization, measured by 20 subconditions excluding blood transfusion. Discrete-time logistic regression was used to estimate associations with long- and short-term heat exposure. Effect modification by maternal characteristics and green space exposure was examined using interaction terms.

**Results:**

There were 3446 SMM cases (0.9%) among 403 602 pregnancies (mean [SD] age, 30.3 [5.7] years). Significant associations were observed with long-term heat exposure during pregnancy and during the third trimester. High exposure (≥80th percentile of the proportions) to extreme heat days during pregnancy and during the third trimester were associated with a 27% (95% CI, 17%-37%; *P* < .001) and 28% (95% CI, 17%-41%; *P* < .001) increase in risk of SMM, respectively. Elevated SMM risks were significantly associated with short-term heatwave exposure under all heatwave definitions. The magnitude of associations generally increased from the least severe (HWD1: daily maximum temperature >75th percentile lasting for ≥2 days; odds ratio [OR], 1.32; 95% CI, 1.17-1.48; *P* < .001) to the most severe heatwave exposure (HWD9: daily maximum temperature >95th percentile lasting for ≥4 days; OR, 2.39; 95% CI, 1.62-3.54; *P* < .001). Greater associations were observed among mothers with lower educational attainment (OR for high exposure to extreme heat days during pregnancy, 1.43; 95% CI, 1.26-1.63; *P* < .001) or whose pregnancies started in the cold season (November through April; OR, 1.37; 95% CI, 1.24-1.53; *P* < .001).

**Conclusions and Relevance:**

In this retrospective cohort study, long- and short-term heat exposure during pregnancy was associated with higher risk of SMM. These results might have important implications for SMM prevention, particularly in a changing climate.

## Key Points

**Question:**

Is maternal environmental heat exposure associated with increased risk of severe maternal morbidity?

**Findings:**

In this cohort study with 403 602 pregnancies from 2008 to 2018 in Southern California, statistically significant associations were observed between both long- and short-term maternal heat exposure during pregnancy and increased risks of severe maternal morbidity.

**Meaning:**

Maternal heat exposure during pregnancy is a potential environmental risk factor for severe maternal morbidity.

## Teaser

This cohort study evaluates associations between maternal heat exposure and risk of severe maternal morbidity.

## Introduction

Severe maternal morbidity (SMM) is considered a near-miss for maternal mortality, referring to severe and unexpected conditions during labor and delivery.^1,2^ Despite improvements in prenatal care coverage and quality due to technological advances (eg, improved screening and treatment for medical conditions during pregnancy and better identification and interventions for risk factors associated with adverse pregnancy outcomes), the prevalence of SMM has continued to increase in the US.^3,4,5,6,7^ The rate of SMM in 2014 was almost 3 times that of 20 years ago.^1^ Some explanations have been proposed, such as improvement in case identification and changes in maternal characteristics (eg, more mothers with an early or advanced age or obesity),^3,8,9,10^ but those proposed factors cannot fully explain the upward trend of SMM.^1^ It is thus imperative to identify more preventable risk factors for SMM,^11^ such as climate-sensitive exposure.

Extreme heat episodes with higher severity have rapidly increased in the past few decades^12^ and have been associated with adverse pregnancy outcomes.^13,14,15^ A recent study identified geographic hotspots with elevated risks of SMM in South Carolina (ie, high-risk SMM clusters).^16^ By differentiating the characteristics between individuals with SMM living in high-risk clusters vs nonclusters, the authors found that individuals exposed to extreme heat during pregnancy were more likely to live in high-risk SMM clusters.^16^ So far, extreme heat has been associated with many adverse obstetric outcomes, including preterm birth, premature rupture of membranes, low birth weight, and stillbirth,^13,17,18,19^ while little evidence is available regarding individual-level SMM risk. Identifying modifiable environmental factors, such as extreme heat, can be critical for minimizing SMM risks.

In recent years, cardiovascular conditions have become a leading cause of pregnancy-related deaths.^3,8,11,20,21^ Existing literature has associated extreme heat exposure with adverse cardiovascular outcomes.^12,22,23,24^ Given the susceptibility of pregnant women, it would also be meaningful to investigate the underlying relationships between heat and maternal cardiovascular conditions with SMM, which may help to explain potential associations between heat and SMM and guide a more targeted intervention for minimizing heat-related SMM risks. In addition, since extreme heat may be increasingly associated with adverse maternal health outcomes in the changing climate, identifying effect modifiers such as maternal characteristics or other environmental factors (eg, residential green space) would provide important information for designing and implementing interventions for SMM.

We conducted a retrospective cohort study to estimate associations between long- and short-term maternal heat exposure and SMM. Furthermore, we examined potential effect modification by maternal characteristics and green space exposure.

## Methods

### Study Population

From 2008 to 2018, we identified 425 722 singleton pregnancies in a large pregnancy cohort from Kaiser Permanente Southern California, an integrated health care organization providing high-quality services throughout Southern California. We obtained detailed information on demographic characteristics, medical histories, self-reported lifestyles, and residential changes throughout pregnancy from KPSC’s electronic health records.^18,25,26^ The gestational age of pregnancies in the KPSC cohort ranged from 20 to 47 weeks and was estimated based on early pregnancy ultrasonography examinations or self-reported last menstrual period (eMethods in Supplement 1). Race and ethnicity data were self-reported and recorded in the social history of electronic health records. The data were reported to account for differences in risk of SMM and to assess potential differences in susceptibility to heat exposure. The study followed the Strengthening the Reporting of Observational Studies in Epidemiology (STROBE) reporting guideline, and was approved by the institutional review board of the KPSC with a waiver for informed consent, as the research was considered minimal risk for participants.

### Outcome Ascertainment

We identified SMM cases during delivery hospitalization based on the US Centers for Disease Control and Prevention SMM index using corresponding 9th and 10th revisions of the *International Classification of Diseases* (*ICD*) codes^27,28^ (eMethods in Supplement 1). The SMM index included 21 indicators shown in eTable 1 in Supplement 1, and a mother with any of those indicators was diagnosed with SMM (denoted as SMM_21_ given 21 indicators included). As the *ICD-9* or *ICD-10* procedure codes cannot provide information about the number of units of blood transfusion and may result in artificial overestimation of SMM_21_ diagnosis, prior studies have measured the SMM rate without blood transfusion.^16,28,29,30^ Following prior studies, we selected SMM_20_, SMM measured without blood transfusion, as the primary outcome in our study. For the secondary outcome, we selected cardiovascular conditions in SMM, denoted as SMM_cardio_. To define this subcategory, we combined cardiovascular conditions with other conditions of similar pathoetiology (eg, eclampsia or cerebrovascular disorders), for a total of 9 conditions (eTable 1 in Supplement 1).

### Heat Exposure Assessment During Pregnancy

We obtained daily maximum temperature data from 2007 to 2018 in Southern California at a 4 km × 4 km resolution from the gridMET data set.^31^ We assigned daily maximum temperature values during pregnancy to each individual based on their geocoded home addresses accounting for residential mobility. We excluded pregnancies if they had less than 75% temperature data available during pregnancy (n = 22 120 [5.2%]) due to missing residential information. We compared the characteristics of included vs excluded pregnancies (eTable 2 and eResults in Supplement 1). For pregnancies with 25% or less missing data (n = 12 906 [3.0%]), we assigned the temperature data of the following home address to the preceding period with missing data.

For long-term heat exposure, we measured the proportions of heat days during pregnancy under 3 definitions of heat days (ie, moderate, high, and extreme). Following recent studies in Southern California focusing on heat exposure,^18,19,32^ we defined the moderate, high, and extreme heat days (denoted as HD_P75, HD_P90, and HD_P95, respectively) as daily maximum temperatures exceeding the 75th, 90th, and 95th percentiles of the time series data from May through September 2007 to 2018 in Southern California, respectively. After checking the distribution of the proportions of heat days, we found that the data were highly and positively skewed. The proportions of moderate, high, and extreme heat days during pregnancy had a skewness of 1.62, 4.73, and 8.43, respectively. Therefore, we dichotomized the exposure variable as low (less than 80th percentile of the proportions) vs high (80th percentile or higher of the proportions) levels.

We examined short-term heatwave exposure during the last gestational week. Nine heatwave definitions (denoted as HWD1-HWD9) were proposed by combining 3 types of heat days (ie, HD_P75, HD_P90, and HD_P95) with 3 durations (ie, ≥2, ≥3, and ≥4 consecutive days).^18,19,32^ We dichotomized short-term exposure to heatwaves as exposed (ie, experiencing at least 1 heatwave) vs unexposed for each heatwave definition.^14,18^

### Green Space Exposure Assessment

We estimated green space exposure based on street view images within a 500 m radius surrounding the residential address at delivery^33,34^ and obtained data from a validated machine-learning model developed in our prior study.^35,36^ In brief, we collected street view images in Southern California from Microsoft Bing Maps Application Programming Interface for each street sampling location with an interval of 200 m. We then estimated the exposure to total green space and 3 different subtypes of green space (ie, trees, low-lying vegetation, and grass) by averaging the proportions of corresponding greenery pixels in all street view images within the 500 m buffer.^35,36^

### Statistical Analysis

Summary statistics were calculated for the characteristics of the study population and exposure variables. We applied the discrete-time logistic regression to examine associations between long- and short-term heat exposure and SMM. As an alternative method to the Cox proportional hazards model, the discrete-time approach is flexible and can be used to estimate associations over the event time based on the proportional hazards assumption or obtain estimates for different time points to relax the assumption.^26,37,38,39^ In this study, we estimated associations based on the proportional hazards assumption by using the discrete-time method in our main analysis and compared the results with that of the Cox proportional hazards model in the sensitivity analysis. We examined associations with long-term heat exposure during the entire pregnancy and by trimester. We examined associations with short-term heatwave exposure during the final gestational week for mothers with deliveries in the hottest period of the year (May to September). Given that the results of short-term heat exposure and SMM_cardio_ may be imprecise due to limited SMM_cardio_ cases in the study period (n = 289), we estimated associations with short-term heat exposure for only the primary outcome, SMM_20_. We selected potential covariates a priori according to existing literature (eMethods in Supplement 1),^16,28^ including maternal age, race and ethnicity, education level, income level, year of delivery, and season of conception. The results were represented by odds ratios (ORs) with corresponding 95% CIs.

We examined effect modification on the multiplicative scale of entire-pregnancy associations for the primary outcome by maternal age, race and ethnicity, education level, income level, smoking, season of conception, and green space exposure using interaction terms.^39^ For the season of conception, we examined associations in the cold season (November to April) vs the warm season (May to October). For green space exposure, we examined effect modification by different levels (high [≥50th] vs low [<50th]) of exposure to total green space, trees, low-lying vegetation, and grass in separate models.

In the sensitivity analysis, we examined associations with SMM_21_ and explored some prevalent SMM subconditions, including blood transfusion (SMM_blood_transf_), disseminated intervascular coagulation (SMM_DIC_), and sepsis (SMM_sepsis_). More analyses conducted to check the robustness of our results are described in eMethods in Supplement 1.

A 2-sided *P* value <.05 was considered statistically significant. All analyses were performed with SAS software version 9.4 (SAS Institute). Adjustment for multiple comparisons was not made for the secondary outcome or any outcome in the sensitivity analysis, and those results should be interpreted as exploratory.

## Results

We included a total of 403 602 pregnancies, with a mean (SD) age of 30.3 (5.7) years; 31 432 included pregnancies (7.8%) were from mothers who were African American, 50 852 (12.6%) who were Asian, 204 817 (50.8%) who were Hispanic, 105 886 (26.2%) who were non-Hispanic White, and who 10 583 (2.6%) were of other races or ethnicities (including Native American Alaskan, Pacific Islander, other unspecified races or ethnicities, and multiple races or ethnicities, consolidated due to the relatively small sample size of each group in the present study) (Table 1). There were 7098 (1.8%) SMM_21_, 3446 (0.9%) SMM_20_, and 684 (0.2%) SMM_cardio_ cases. The descriptive statistics of exposure variables are shown in Tables 2 and 3. The median (IQR) percentages of moderate (HD_P75), high (HD_P90), and extreme (HD_P95) heat days during pregnancy were 3.93% (0.73%-11.07%), 0.72% (0.00%-2.87%), and 0.00% (0.00%-1.06%), respectively.

**Table 1.** Descriptive Statistics of the Study Population Based on SMM Status, 2008 to 2018

Characteristic	Pregnancies, No. (%)				
	Total pregnancies (N = 403 602)	Non-SMM (n = 396 504)	SMM_21_ (n = 7098)	SMM_20_ (n = 3446)	SMM_cardio_ (n = 684)
Maternal age, y					
<25	76 290 (18.90)	74 981 (18.91)	1309 (18.44)	575 (16.69)	130 (19.01)
25-34	239 707 (59.39)	235 838 (59.48)	3869 (54.51)	1857 (53.89)	344 (50.29)
≥35	87 605 (21.71)	85 685 (21.61)	1920 (27.05)	1014 (29.43)	210 (30.70)
Race and ethnicity^a^					
African American	31 432 (7.79)	30 660 (7.73)	772 (10.88)	413 (11.98)	81 (11.84)
Asian	50 852 (12.60)	49 867 (12.58)	985 (13.88)	482 (13.99)	92 (13.45)
Hispanic	204 817 (50.75)	201 347 (50.78)	3470 (48.89)	1598 (46.37)	310 (45.32)
Non-Hispanic White	105 886 (26.24)	104 223 (26.29)	1663 (23.43)	855 (24.81)	180 (26.32)
Other^b^	10 583 (2.62)	10 376 (2.62)	207 (2.92)	97 (2.81)	21 (3.07)
Missing	32 (0.01)	31 (0.01)	1 (0.01)	1 (0.03)	0
Education level					
<College	123 416 (30.58)	121 331 (30.60)	2085 (29.37)	1024 (29.72)	204 (29.82)
≥College	272 168 (67.43)	267 351 (67.43)	4817 (67.86)	2322 (67.38)	458 (66.96)
Missing	8018 (1.99)	7822 (1.97)	196 (2.76)	100 (2.90)	22 (3.22)
Income level, $					
Mean (SD)	59 871 (21 835)	59 889 (21 834)	58 853 (21 881)	59 434 (22 363)	58 721 (21 148)
Missing	1237 (0.31)	1217 (0.31)	20 (0.28)	10 (0.29)	2 (0.29)
Insurance type					
Medicaid	38 500 (9.54)	37 724 (9.51)	776 (10.93)	383 (11.11)	73 (10.67)
Other	359 983 (89.19)	353 753 (89.22)	6230 (87.77)	3023 (87.72)	606 (88.60)
Missing	5119 (1.27)	5027 (1.27)	92 (1.30)	40 (1.16)	5 (0.73)
Season of conception					
Cool (November-April)	205 781 (50.99)	202 122 (50.98)	3659 (51.55)	1723 (50.00)	344 (50.29)
Warm (May-October)	197 821 (49.01)	194 382 (49.02)	3439 (48.45)	1723 (50.00)	340 (49.71)
BMI					
Underweight (<18.5)	9587 (2.38)	9403 (2.37)	184 (2.59)	74 (2.15)	14 (2.05)
Normal weight (18.5-24.9)	171 348 (42.45)	168 375 (42.46)	2973 (41.89)	1409 (40.89)	272 (39.77)
Overweight (25.0-29.9)	112 861 (27.96)	110 900 (27.97)	1961 (27.63)	959 (27.83)	170 (24.85)
Obese (≥30.0)	107 751 (26.70)	105 818 (26.69)	1933 (27.23)	978 (28.38)	221 (32.31)
Missing	2055 (0.51)	2008 (0.51)	47 (0.66)	26 (0.75)	7 (1.02)
Parity					
Primiparous	164 837 (40.84)	161 499 (40.73)	3338 (47.03)	1563 (45.36)	348 (50.88)
Multiparous	236 771 (58.66)	233 112 (58.79)	3659 (51.55)	1833 (53.19)	327 (47.81)
Missing	1994 (0.49)	1893 (0.48)	101 (1.42)	50 (1.45)	9 (1.32)
Smoking status					
Never smoked	335 882 (83.22)	329 944 (83.21)	5938 (83.66)	2877 (83.49)	563 (82.31)
Past smoking	46 949 (11.63)	46 185 (11.65)	764 (10.76)	375 (10.88)	77 (11.26)
Smoked during pregnancy	20 746 (5.14)	20 350 (5.13)	396 (5.58)	194 (5.63)	44 (6.43)
Missing	25 (0.01)	25 (0.01)	0	0	0
Medical conditions					
Preterm birth	31 319 (7.76)	29 866 (7.53)	1453 (20.47)	866 (25.13)	233 (34.06)
Gestational diabetes^c^	41 137 (10.19)	40 374 (10.18)	763 (10.75)	379 (11.00)	78 (11.40)
Gestational hypertension	19 057 (4.72)	18 528 (4.67)	529 (7.45)	272 (7.89)	89 (13.01)
Preexisting diabetes	5382 (1.33)	5162 (1.30)	220 (3.10)	131 (3.80)	35 (5.12)
Chronic hypertension	13 886 (3.44)	13 439 (3.39)	447 (6.30)	264 (7.66)	86 (12.57)

Abbreviations: BMI, body mass index (calculated as weight in kilograms divided by height in meters squared); SMM, severe maternal morbidity; SMM_20_, SMM measured without the blood product transfusion; SMM_21_, SMM measured with all 21 indicators; SMM_cardio_, the cardiovascular-related subcategory in SMM.

^a^ Race and ethnicity data were self-reported and recorded in the social history of electronic health records. The data were reported to account for differences in risk of SMM and to assess potential differences in susceptibility to heat exposure.

^b^ Other included Native American Alaskan, Pacific Islander, other unspecified races or ethnicities, and multiple races or ethnicities, consolidated due to the relatively small sample size of each group in the present study.

^c^ The missing numbers of gestational diabetes among total pregnancies, non-SMM cases, SMM_21_ cases, SMM_20_ cases, and SMM_cardio_ cases are 26 440 (6.55%), 25 680 (6.48%), 760 (10.71%), 398 (11.55%), and 95 (13.89%), respectively.

**Table 2.** Descriptive Statistics of Heat and Green Space Exposures During the Entire Pregnancy for the Study Population Based on SMM Status, 2008 to 2018

	Total pregnancies (N = 403 602)	Non-SMM (n = 396 504)	SMM_21_ (n = 7098)	SMM_20_ (n = 3446)	SMM_cardio_ (n = 684)
Proportions of heat days, median (IQR), %					
HD_P75	3.93 (0.73-11.07)	3.94 (0.73-11.11)	3.52 (0.72-10.83)	4.00 (0.74-11.91)	3.99 (0.72-13.60)
HD_P90	0.72 (0.00-2.87)	0.72 (0.00-2.87)	0.71 (0.00-2.87)	0.75 (0.00-3.24)	0.74 (0.00-3.92)
HD_P95	0.00 (0.00-1.06)	0.00 (0.00-1.06)	0.00 (0.00-1.07)	0.00 (0.00-1.24)	0.35 (0.00-1.41)
Green space exposure, mean (SD), %					
Total green space	25.24 (4.09)	25.24 (4.09)	25.26 (4.16)	25.17 (4.14)	24.98 (3.91)
Trees	15.34 (4.22)	15.34 (4.22)	15.37 (4.25)	15.20 (4.34)	15.02 (4.21)
Low-lying vegetation	4.58 (1.46)	4.58 (1.46)	4.59 (1.46)	4.59 (1.44)	4.57 (1.44)
Grass	5.33 (1.48)	5.33 (1.48)	5.31 (1.48)	5.37 (1.52)	5.39 (1.62)
Missing, No. (%)	1026 (0.25)	998 (0.25)	28 (0.39)	20 (0.58)	4 (0.58)

Abbreviations: HD_P75, moderate heat days exceeding the 75th daily maximum temperature percentile; HD_P90, high heat days exceeding the 90th daily maximum temperature percentile; HD_P95, extreme heat days exceeding the 95th daily maximum temperature percentile; SMM, severe maternal morbidity; SMM_20_, SMM measured without the blood product transfusion; SMM_21_, SMM measured with all 21 indicators; SMM_cardio_, the cardiovascular-related subcategory in SMM.

**Table 3.** Characteristics of Heatwave Definitions and Numbers of Pregnancies Exposed to Heatwaves From May Through September 2008 to 2018

Definition	Cutoff values	No. (%)	
		Non-SMM^a^	SMM_20_^b^
HWD1 (DMT > 75th percentile lasting for ≥2 d)^c^	35.05 °C	42 015 (24.62)	418 (26.46)
HWD2 (DMT > 75th percentile lasting for ≥3 d)^c^	35.05 °C	28 912 (16.94)	308 (19.49)
HWD3 (DMT > 75th percentile lasting for ≥4 d)^c^	35.05 °C	18 685 (10.95)	209 (13.23)
HWD4 (DMT > 90th percentile lasting for ≥2 d)^d^	38.15 °C	13 672 (8.01)	159 (10.06)
HWD5 (DMT > 90th percentile lasting for ≥3 d)^d^	38.15 °C	7365 (4.32)	84 (5.32)
HWD6 (DMT > 90th percentile lasting for ≥4 d)^d^	38.15 °C	4055 (2.38)	48 (3.04)
HWD7 (DMT > 95th percentile lasting for ≥2 d)^e^	39.95 °C	6087 (3.57)	71 (4.49)
HWD8 (DMT > 95th percentile lasting for ≥3 d)^e^	39.95 °C	2690 (1.58)	37 (2.34)
HWD9 (DMT > 95th percentile lasting for ≥4 d)^e^	39.95 °C	1383 (0.81)	26 (1.65)

Abbreviations: DMT, daily maximum temperature; HWD, heatwave definition; SMM, severe maternal morbidity; SMM_20_, SMM measured without the blood product transfusion.

^a^ The total number of non-SMM cases from May through September 2008 to 2018 was 170 679.

^b^ The total number of SMM_20_ cases from May through September 2008 to 2018 was 1580.

^c^ HWD1, HWD2, and HWD3 are defined as daily maximum temperature >75th percentile lasting for ≥2, ≥3, and ≥4 days, respectively.

^d^ HWD4, HWD5, and HWD6 are defined as daily maximum temperature >90th percentile lasting for ≥2, ≥3, and ≥4 days, respectively.

^e^ HWD7, HWD8, and HWD9 are defined as daily maximum temperature >95th percentile lasting for ≥2, ≥3, and ≥4 days, respectively.

### Long-Term Heat Exposure and Risk of SMM

The adjusted ORs and corresponding CIs of SMM_20_ and SMM_cardio_ associated with high exposure to heat days (≥80th percentile of proportions) are shown in Table 4. Significantly increased risk of SMM_20_ and SMM_cardio_ were mainly associated with heat exposure during the entire pregnancy and the third trimester. The magnitude of associations was higher for more severe heat exposure. High exposure to HD_P95 during pregnancy was associated with a 27% increase in SMM_20_ risk (95% CI, 17%-37%; *P* < .001). We observed a higher magnitude of associations for SMM_cardio_ than SMM_20_. The highest risk of SMM_cardio_ was associated with high exposure to HD_P95 during the third trimester (OR, 1.51; 95% CI, 1.22-1.87; *P* < .001).

**Table 4.** Adjusted Odds Ratios (ORs) With 95% CIs of SMM_20_ and SMM_cardio_ Associated With High Exposure to Heat Days (≥80th Percentile of Proportions)

	Exposure window			
	First trimester^a^	Second trimester^a^	Third trimester^a^	Entire pregnancy^a^
**SMM_20_, OR (95% CI)**				
HD_P75	1.07 (0.98-1.17)	1.00 (0.92-1.09)	1.16 (1.06-1.27)	1.14 (1.05-1.24)
HD_P90	1.04 (0.95-1.13)	0.98 (0.90-1.06)	1.27 (1.17-1.39)	1.17 (1.08-1.27)
HD_P95	1.08 (0.98-1.18)	1.02 (0.93-1.11)	1.28 (1.17-1.41)	1.27 (1.17-1.37)
**SMM_cardio_, OR (95% CI)**				
HD_P75	1.16 (0.95-1.42)	1.15 (0.96-1.38)	1.15 (0.93-1.41)	1.39 (1.16-1.65)
HD_P90	1.21 (0.99-1.46)	1.09 (0.90-1.31)	1.33 (1.08-1.63)	1.50 (1.26-1.78)
HD_P95	1.34 (1.09-1.64)	1.16 (0.95-1.41)	1.51 (1.22-1.87)	1.48 (1.25-1.76)

Abbreviations: HD_P75, moderate heat days exceeding the 75th daily maximum temperature percentile; HD_P90, high heat days exceeding the 90th daily maximum temperature percentile; HD_P95, extreme heat days exceeding the 95th daily maximum temperature percentile; SMM_20_, severe maternal morbidity measured without the blood product transfusion; SMM_cardio_, the cardiovascular-related subcategory in severe maternal morbidity.

^a^ Models adjusted for maternal age, race and ethnicity, education level, income level, year of delivery, and season of conception.

### Short-Term Heatwave Exposure and Risk of SMM

The Figure shows associations between exposure to heatwaves during the last gestational week and SMM_20_. All associations were significant under different heatwave definitions. The magnitude of associations generally increased from the least severe (HWD1: daily maximum temperature >75th percentile lasting for ≥2 days; OR, 1.32; 95% CI, 1.17-1.48; *P* < .001) to the most severe heatwave exposure (HWD9: daily maximum temperature >95th percentile lasting for ≥4 days; OR, 2.39; 95% CI, 1.62-3.54; *P* < .001).

**Figure.**
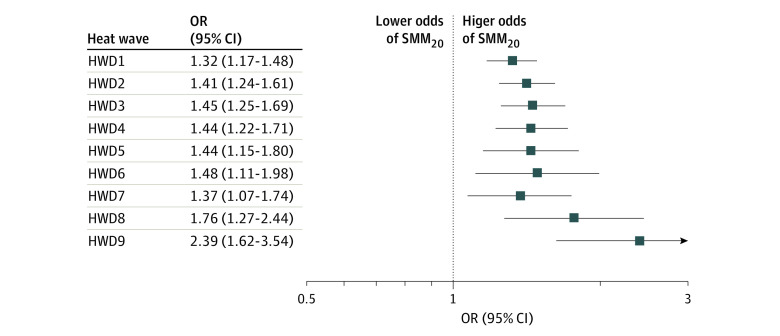
Adjusted Odds Ratios (ORs) With 95% CIs of Severe Maternal Morbidity Associated With Exposure to Heatwaves Under Different Definitions During the Last Gestational Week Models adjusted for maternal age, race and ethnicity, education level, income level, and year of delivery. HWD1, HWD2, and HWD3 are defined as daily maximum temperature >75th percentile lasting for ≥2, ≥3, and ≥4 days, respectively. HWD4, HWD5, and HWD6 are defined as daily maximum temperature >90th percentile lasting for ≥2, ≥3, and ≥4 days, respectively. HWD7, HWD8, and HWD9 are defined as daily maximum temperature >95th percentile lasting for ≥2, ≥3, and ≥4 days, respectively. HWD indicates heatwave definition; SMM_20_, severe maternal morbidity measured without the blood product transfusion.

### Effect Modification by Maternal Characteristics and Green Space Exposure

We observed significant interactions between long-term heat exposure and 2 maternal characteristics, that is, education level and season of conception (eTable 3 in Supplement 1). We observed significantly higher associations between heat exposure and SMM_20_ among mothers who did not attend college or whose pregnancies began in the cold season than those with a higher academic degree or starting pregnancies in the warm season, respectively. Although the interaction of other characteristics was not statistically significant, we observed consistently higher associations among mothers younger than 25 years or 35 years and older, from a Hispanic racial or ethnic background, with a lower income level, who smoked, or who had less exposure to trees or grass (eTables 3 and 4 in Supplement 1).

### Sensitivity Analyses

We observed associations of SMM_21_ with only short-term heat exposure (eTables 5 and 6 in Supplement 1). Increased risks of SMM_sepsis_ were consistently associated with long- and short-term heat exposure. We observed negative associations of SMM_blood_transf_ with long-term heat exposure. The results of other sensitivity analyses were comparable to those of our main analyses (eResults and eTables 7 and 8 in Supplement 1).

## Discussion

In this large retrospective cohort study, long- and short-term maternal exposure to environmental heat was significantly associated with higher SMM risks during delivery hospitalization. We also identified effect modification by the education level and season of conception.

There is abundant literature documenting potential risk factors for SMM, such as early or advanced age, lower socioeconomic status, and obesity.^29,40,41,42,43^ To provide new insights for SMM prevention, we focused on environmental risk factors that have not been examined previously. We observed that experiencing more heat days during pregnancy was associated with increased risks of SMM, where risks increased as heat exposure became more extreme. Associations observed in the third trimester indicated that late pregnancy might be a more sensitive window to environmental heat. We noticed that women who started pregnancy in the cold season were more vulnerable to heat exposure, likely because they would experience their late pregnancy in the hottest period in Southern California (May-September). By comparison, those who started pregnancy in the warm season may experience fewer heat days in their third trimester. Associations with heatwaves observed at the end of pregnancy also support the heat impact in late pregnancy.

Following existing research on SMM,^16,28,29,30^ we estimated associations with SMM_20_ (SMM measured without blood transfusion) and with SMM_21_ (SMM measured with all indicators). We found that associations with SMM_21_ were much weaker compared with those of SMM_20_, especially for associations with long-term heat exposure. We further explored associations with SMM_blood_transf_ and found negative associations with long-term heat exposure. Preeclampsia is one of the most frequent causes of uterine atony and postpartum hemorrhage,^44,45^ which can result in blood transfusion during delivery hospitalization.^46^ A previous study reported that cold ambient temperature was positively associated with, while hot temperature was negatively associated with, the incidence of preeclampsia,^47^ which might be a potential explanation for negative associations between heat exposure and SMM_blood_transf_. Physiologic evidence also suggests a positive association between a cold pressor stimulus as well as skin cooling and preeclampsia.^48,49^ Without information about the number of blood transfusion units, the diagnosis of SMM_blood_transf_ may not accurately reflect the severity of maternal conditions, which can lead to bias in our results. Additional physiologic, clinical, and observational experience is needed to elucidate the associations between environmental heat, blood transfusion during delivery, and circumstances in pregnancy indicating the need for blood.

About one-third of SMM indicators are associated with cardiovascular, cerebrovascular, or hypertensive conditions, which usually share similar risk factors and pathogenesis.^50,51,52^ We observed a higher magnitude of associations with long-term heat exposure and SMM_cardio_ compared to associations with SMM_20_, although results for SMM_cardio_ were less precise with wider 95% CIs due to fewer cases. It suggests that the cardiovascular subconditions of SMM may play a critical role in associations between heat exposure and SMM. Our findings were supported by previously reported associations between heat exposure and cardiovascular-related outcomes with postulated mechanisms, such as leading to volume depletion and electrolyte imbalance, inducing inflammation and hypercoagulable states, and increasing heart rate and metabolic state.^22,23,24,53,54,55,56^ We also looked at other prevalent SMM subconditions and observed significant associations for SMM_sepsis_. One possible explanation was that the hypovolemia resulting from heat exposure may promote localized infection to sepsis.^57^ More in-depth studies can help to understand associations with different SMM subconditions.

Mothers younger than 25 years or 35 years and older might be more susceptible to heat-related SMM risks. Higher risks of obstetric problems associated with adolescent and advanced-age pregnancies have been well recognized. Adolescent mothers are more likely to be in unfavorable social or physical conditions^58^ and may have limited knowledge or awareness to protect themselves against extreme heat. Pregnancy complications and risk factors can be more common among older mothers, possibly due to the physiological changes from aging, which may exacerbate heat effects.^58,59,60^

Racial and ethnic disparities in SMM prevalence are known to persist.^61,62^ Even though we did not observe a statistically significant effect modification between heat and race and ethnicity in associations between heat exposure and risk of SMM, we observed a higher magnitude of associations in Hispanic mothers. Prior studies demonstrated that African American women can be disproportionately burdened by SMM risks, followed by Hispanic women and other racial or ethnic minoritized groups.^29,41,42,63,64,65^ Due to a relatively small sample size of the African American pregnant population in our study, results for this group were less precise compared with others. To interpret racial and ethnic heterogeneity, researchers have called for considering race as a socially defined construct.^61,66^ We observed higher proportions of mothers with a lower education or income level in our cohort for African American and Hispanic populations compared with Asian or non-Hispanic White mothers. Moreover, we found significantly higher associations between heat exposure and risk of SMM among mothers with a lower education level. Our observation of worse health outcomes among women with lower socioeconomic status may reflect the broader impacts of the persistent and pervasive social injustice issues, including higher adverse exposure levels, more cumulative stressors, more underlying health conditions, and lack of resources and opportunities among these vulnerable populations.^67^

Green space is a potential heat mitigation strategy. Mothers living with more trees or grass around their residences tended to have lower heat-related SMM risks, even though the effect modification was not statistically significant. Besides reducing heat by providing shade from trees, studies have shown that more social support and physical activity induced by residential green space can lead to better health conditions.^36,68,69,70,71^ Our results may have potential implications for increasing adaptability to extreme heat.

To our knowledge, this is the first study providing evidence of associations between heat exposure during pregnancy and individual-level SMM risk. We also explored heat-related risks of cardiovascular and other subconditions of SMM. Other strengths included the large and diverse pregnancy cohort, the high-quality and rich clinical databases with detailed information on residential mobility during pregnancy, and an extensive analysis of effect modification by maternal characteristics as well as green space exposure.

### Limitations

Some limitations should be acknowledged. First, we examined only ambient temperature and did not consider individual-level time-activity patterns (eg, time indoors or in the workplace) or adaptation behaviors (eg, self-dousing or using air conditioning or fans) due to data unavailability, which may have led to exposure misclassification and biased estimated associations in either direction. Second, we only considered SMM during delivery hospitalization and did not have data during postpartum hospitalizations, which may have led to an underestimation of the overall SMM cases and biased results in either direction.^5^ Third, by using gridded surface air temperature data at a 4-km resolution, we were unable to capture the microscale influences of urban heat islands, which may deserve further investigation.^18^ Fourth, the participants in our study were from a single health care organization in Southern California, which limits the generalizability of our findings. Further studies in other regions with various climates and diverse populations are warranted. Residual confounding resulting from other factors that were not controlled may also exist in this study.

## Conclusions

Long- and short-term maternal heat exposure during pregnancy was associated with increased risks of SMM in this study. Health disparity existed among mothers with different education levels. These results indicate the potential benefit of targeted interventions to reduce the risk of SMM by mitigating maternal heat exposure, especially among mothers with low socioeconomic status.

## Notes

Supplement 1.
eMethods

eResults
**eTable 1.** Frequencies of severe maternal morbidity indicators at delivery hospitalization in the study population, 2008 to 2018 **eTable 2.** Characteristics of included vs. excluded pregnancies **eTable 3.** Effect modification in associations between long-term heat exposure during the entire pregnancy and severe maternal morbidity by maternal characteristics estimated using interaction terms **eTable 4.** Effect modification in associations between long-term heat exposure during the entire pregnancy and severe maternal morbidity by green space exposure estimated using interaction terms **eTable 5.** Adjusted odds ratios (ORs) with 95% CIs of other severe maternal morbidity subconditions associated with long-term heat exposure during the entire pregnancy **eTable 6.** Adjusted odds ratios (ORs) with 95% CIs of other severe maternal morbidity subconditions associated with exposure to heatwaves under different definitions during the last gestational week **eTable 7.** Estimated risks of severe maternal morbidity associated with long-term heat exposure during the entire pregnancy in the sensitivity analysis **eTable 8.** Estimated risks of severe maternal morbidity associated with exposure to heatwaves under different definitions during the last gestational week in the sensitivity analysis

Supplement 2.
Data Sharing Statement
