# Supplementary material for: Analysis of Heat Exposure During Pregnancy and Severe Maternal Morbidity
Source: JAMA Netw Open. 2023 Sep 7;6(9):e2332780. doi: 10.1001/jamanetworkopen.2023.32780 (PMC10485728; doi:10.1001/jamanetworkopen.2023.32780)
Supplement: Supplement 1. — eMethods eResults eTable 1. Frequencies of severe maternal morbidity indicators at delivery hospitalization in the study population, 2008 to 2018 eTable 2. Characteristics of included vs. excluded pregnancies eTable 3. Effect modification in associations between long-term heat exposure during the entire pregnancy and severe maternal morbidity by maternal characteristics estimated using interaction terms eTable 4. Effect modification in associations between long-term heat exposure during the entire pregnancy and severe maternal morbidity by green space exposure estimated using interaction terms eTable 5. Adjusted odds ratios (ORs) with 95% CIs of other severe maternal morbidity subconditions associated with long-term heat exposure during the entire pregnancy eTable 6. Adjusted odds ratios (ORs) with 95% CIs of other severe maternal morbidity subconditions associated with exposure to heatwaves under different definitions during the last gestational week eTable 7. Estimated risks of severe maternal morbidity associated with long-term heat exposure during the entire pregnancy in the sensitivity analysis eTable 8. Estimated risks of severe maternal morbidity associated with exposure to heatwaves under different definitions during the last gestational week in the sensitivity analysis [file jamanetwopen-e2332780-s001.pdf]

Supplemental Online Content

Jiao A, Sun Y, Avila C, et al. Analysis of heat exposure during pregnancy and severe maternal morbidity. *JAMA Netw Open*. 2023;6(9):e2332780. doi:10.1001/jamanetworkopen.2023.32780

eMethods

eResults

eTable 1. The frequency and percentage of severe maternal morbidity (SMM) indicators at delivery hospitalization

eTable 2. The characteristics of included vs. excluded subjects

eTable 3. The effect modification in associations between SMM20 and exposure to heat days during the entire pregnancy by maternal characteristics estimated using interaction terms

eTable 4. The effect modification in associations between SMM20 and exposure to heat days during the entire pregnancy by green space exposure estimated using interaction terms

eTable 5. The estimated risks of SMM20 with 95% confidence intervals (CIs) associated with the high exposure to heat days during the entire pregnancy examined in the sensitivity analysis

eTable 6. The estimated risks of SMM20 with 95% confidence intervals (CIs) associated with heatwaves under different definitions during the last gestational week in the sensitivity analysis

eTable 7. The adjusted odds ratios (ORs) with 95% confidence intervals (CIs) of other SMM outcomes associated with the high exposure to heat days (≥ 80th) during the entire pregnancy

eTable 8. The adjusted odds ratios (ORs) with 95% confidence intervals (CIs) of other SMM outcomes associated with heatwaves under different definitions during the last gestational week

This supplemental material has been provided by the authors to give readers additional information about their work.

© 2023 Jiao A et al. *JAMA Network Open.*

eMethods

Study population

The gestational age was recorded in KPSC's electronic health records (EHRs) and ranged from 20 to 47 weeks. It was largely estimated based on first-trimester sonographic estimates for mothers who had early pregnancy ultrasound examinations [1-3]. A few cases (about 5%) were based on self-reported last menstrual period (LMP) for mothers without sonographic estimates. Pregnancies with gestational ages < 20 weeks were defined as miscarriages and excluded. Estimates based on self-reported LMP may have resulted in inaccurate estimations of gestational age and extreme outliers. Thus, extremely long pregnancies with gestational ages > 47 weeks were further excluded.

Outcome Ascertainment

The EHRs of KPSC contain clinical diagnoses and procedural codes as well as data from laboratory records related to maternal medical comorbidities and obstetrical complications of the index pregnancy to identify women with SMM at the time of delivery admission. In the current study, the clinical diagnosis, procedural, and laboratory record dates that were used to determine the SMM diagnosis dates correspond with the delivery dates.

Covariates

Covariates included in the data analysis were specified a priori based on previous studies. Through the literature review [4-24], we chose to adjust for maternal age, race/ethnicity, and socioeconomic status (SES) in our main analysis. For SES, we used educational attainment and income level as the proxy, both of which are commonly associated with SMM. As our study period is from 2008 to 2018, we further adjusted for the year of delivery to control the potential long-term time trend in associations [2,3,25]. Besides, for associations with long-term heat exposure, we further adjusted for the season of conception to control the potential seasonal trend. In the sensitivity analysis, we further adjusted for other potential confounders, including insurance type, pre-pregnancy body mass index (BMI), parity, preterm birth, pregnancy complications (including gestational diabetes and gestational hypertension), and pre-existing medical conditions (including pre-existing diabetes or chronic hypertension). Data on all covariates were obtained from the KPSC EHRs.

Sensitivity analysis

We conducted sensitivity analyses for associations with the primary outcome and heat exposure during the entire pregnancy and the last gestational week to check the robustness of our results: a) We performed unadjusted models; b) We performed models by further adjusting for different sets of confounders, including insurance type, pre-pregnancy BMI, parity, preterm birth, pregnancy complications, and maternal pre-existing medical conditions; c) We included the county or zip code of residence as a random effect to account for potential spatial clustering of SMM cases; d) We accounted for the potential effect of mothers who had multiple deliveries by including only the first delivery of each mother during the study period. In the entire cohort (n = 403,602), the number of included pregnancies was 300,748 (74.52%), from 213,346 mothers with one delivery and 87,402 mothers with multiple deliveries, respectively; e) We restricted our study population to pregnancies with gestational ages of 20-43 weeks (n = 403,541, 99.98%) to account for the potential impact of extreme outliers of gestational age on our findings; f) We applied daily maximum heat index incorporating temperature and relative humidity as an alternative temperature metric; g) We used the Cox proportional hazards model as an alternative method; h) We used the existing home address to fill in the preceding missing period for subjects with missing residential data and then performed data analyses for all subjects (n = 425,722). Finally, for associations with heat exposure during the entire pregnancy, we used the proportion of heat days as a continuous exposure variable in the model.

© 2023 Jiao A et al. *JAMA Network Open.*

eResults

We excluded 22,120 (5.2%) pregnancies in the main analysis as they had < 75% exposure data available during pregnancy due to missing residential information. We compared the characteristics between included (n = 403,602) vs. excluded subjects (n = 22,120). We observed that subjects who were younger, self-identified as Asian or Hispanic, with lower educational attainment or lower income level, or with Medicaid insurance were more likely to have missing residential information during pregnancy (eTable 2). The electronic health records (EHRs) of KPSC document residential mobility during pregnancy of women who are members of KPSC. Low-income individuals and those with publicly funded insurance (e.g., Medicaid) tend to be highly mobile and may not maintain a KPSC membership as well as a long-term residential address.

The results of sensitivity analyses for associations between SMM_{20} and long- and short-term heat exposure are shown in eTable 5 and eTable 6, respectively. We observed similar associations from the unadjusted models compared to those from the adjusted models in the main analysis. Models further adjusted for different sets of covariates revealed comparable results to that of our main analysis. After controlling pregnancy complications, the magnitude of associations was slightly attenuated, and some associations were statistically insignificant. Preterm birth and pregnancy complications, such as gestational diabetes or gestational hypertension, might be potential mediators of associations between heat exposure and SMM, which can be estimated in future research. After controlling spatial clustering, though the magnitude of associations was slightly attenuated and some associations with short-term heatwave exposures were statistically insignificant, the results were largely robust, and our conclusions did not change. We observed similar results before vs. after controlling for multiple deliveries. Restricting the study population to pregnancies with a gestational age of 20-43 weeks changed the results minimally. We observed slightly attenuated associations between heat exposure and SMM by using the daily maximum heat index as an alternative temperature metric. The Cox proportional hazards models showed similar results compared to our main analysis using the discrete-time regression. After filling in missing residential information, the model for all subjects showed similar results compared to that of our main analysis. By using the continuous exposure variable, we observed significant associations between long-term heat exposure and SMM. The odds ratios (ORs) of SMM_{20} per 10% increase in the proportion of moderate, high, and extreme heat days during the pregnancy were 1.12 (95% CI: 1.08-1.17; $P < .001$), 1.37 (95% CI: 1.28-1.46; $P < .001$), and 1.55 (95% CI: 1.41-1.69; $P < .001$), respectively.

© 2023 Jiao A et al. *JAMA Network Open.*

eTable 1 The frequency and percentage of severe maternal morbidity (SMM) indicators at delivery hospitalization.

SMM indicators [a]	Frequency (percentage, %)
Air and thrombotic embolism	87 (1.17)
Aneurysm [b]	6 (0.08)
Amniotic fluid embolism	25 (0.33)
Acute myocardial infarction [b]	8 (0.11)
Adult respiratory distress syndrome	254 (3.40)
Acute renal failure	353 (4.73)
Blood transfusion	4,603 (61.64)
Cardiac arrest/ventricular fibrillation [b]	30 (0.40)
Conversion of cardiac rhythm [b]	57 (0.76)
Disseminated intravascular coagulation	1,542 (20.65)
Eclampsia [b]	214 (2.87)
Hysterectomy	425 (5.69)
Heart failure /arrest during surgery or procedure [b]	38 (0.51)
Pulmonary edema/acute heart failure [b]	148 (1.98)
Puerperal cerebrovascular disorders [b]	134 (1.79)
Severe anesthesia complications	49 (0.66)
Sepsis	580 (7.77)
Sickle cell disease with crisis	21 (0.28)
Shock [b]	146 (1.96)
Temporary tracheostomy	5 (0.07)
Ventilation	305 (4.08)

[a] There were 7,467 (1.75%) SMM cases measured with all 21 indicators in the KPSC pregnancy cohort (n = 425,722).
[b] The indicator included in the cardiovascular-related conditions in SMM (SMM_{cardio}).

© 2023 Jiao A et al. *JAMA Network Open.*

eTable 2 The characteristics of included vs. excluded subjects.

Characteristics	Pregnancies included (n = 403,602)	Pregnancies excluded (n = 22,120)	*P* value
Maternal age, years (n, %)			<.001
< 25	76,290 (18.90)	5,832 (26.37)	
25-34	239,707 (59.39)	13,112 (59.28)	
≥ 35	87,605 (21.71)	3,176 (14.36)	
Race/Ethnicity (n, %)			<.001
African American	31,432 (7.79)	1,013 (4.58)	
Asian	50,852 (12.60)	3,130 (14.15)	
Hispanic	204,817 (50.75)	12,002 (54.26)	
Non-Hispanic White	105,886 (26.24)	5,376 (24.30)	
Others [a]	10,583 (2.62)	595 (2.69)	
Missing	32 (0.01)	4 (0.02)	
Education level (n, %)			<.001
Less than college	123,416 (30.58)	8,519 (38.51)	
College or higher	272,168 (67.43)	13,008 (58.81)	
Missing	8,018 (1.99)	593 (2.68)	
Income level, US Dollars			<.001
Mean ± SD	59,871 (21,835)	57,119 (21,364)	
Missing (n, %)	1,237 (0.31)	114 (0.52)	
Insurance type			<.001
Medicaid	38,500 (9.54)	2,372 (10.72)	
Other	359,983 (89.19)	19,486 (88.09)	
Missing	5,119 (1.27)	262 (1.18)	

[a] This racial and ethnic group includes Native American Alaskan, Pacific Islander, other unspecified races/ethnicities, and multiple races/ethnicities.

© 2023 Jiao A et al. *JAMA Network Open.*

eTable 3 The effect modification in associations between SMM_{20} and exposure to heat days during the entire pregnancy by maternal characteristics estimated using interaction terms.

	HD_P75 [a]		HD_P90 [a]		HD_P95 [a]	
	ORs (95% CIs)	*P* value [b]	ORs (95% CIs)	*P* value [b]	ORs (95% CIs)	*P* value [b]
Maternal age						
< 25	1.170 (0.972-1.408)	0.39	1.308 (1.092-1.566)	0.12	1.318 (1.101-1.579)	0.12
25-34	1.084 (0.969-1.213)		1.087 (0.974-1.214)		1.180 (1.060-1.313)	
≥ 35	1.237 (1.056-1.450)		1.270 (1.090-1.479)		1.419 (1.226-1.641)	
Race/Ethnicity						
African American	0.974 (0.761-1.248)	0.08	1.133 (0.894-1.436)	0.38	1.202 (0.951-1.519)	0.90
Asian	0.972 (0.739-1.278)		1.046 (0.809-1.352)		1.250 (0.991-1.576)	
Hispanic	1.250 (1.115-1.401)		1.269 (1.135-1.420)		1.289 (1.154-1.440)	
Non-Hispanic White	1.131 (0.962-1.330)		1.098 (0.935-1.288)		1.289 (1.106-1.503)	
Others [c]	0.688 (0.389-1.216)		0.934 (0.564-1.547)		1.025 (0.630-1.668)	
Education level						
Less than college	1.267 (1.106-1.451)	0.06	1.334 (1.168-1.525)	0.02	1.431 (1.255-1.632)	0.02
College or higher	1.073 (0.967-1.191)		1.095 (0.990-1.212)		1.188 (1.078-1.310)	
Income level						
< 50th	1.209 (1.081-1.352)	0.13	1.245 (1.116-1.389)	0.13	1.320 (1.186-1.469)	0.28
≥ 50th	1.064 (0.942-1.202)		1.099 (0.977-1.237)		1.211 (1.081-1.357)	
Smoking status						
Nonsmoker	1.129 (1.031-1.236)	0.62	1.153 (1.055-1.259)	0.30	1.232 (1.130-1.342)	0.11
Smoker [d]	1.192 (0.978-1.454)		1.288 (1.062-1.561)		1.458 (1.210-1.756)	
Season of conception						
Cool (November-April)	1.242 (1.117-1.381)	0.01	1.294 (1.165-1.438)	0.01	1.373 (1.237-1.525)	0.02
Warm (May-October)	1.000 (0.875-1.143)		1.028 (0.906-1.167)		1.145 (1.016-1.290)	

Abbreviations: CI, confidence interval; HD_P75, moderate heat days exceeding the 75th daily maximum temperature percentile; HD_P90, high heat days exceeding the 90th daily maximum temperature percentile; HD_P95, extreme heat days exceeding the 95th daily maximum temperature percentile; OR, odds ratio; SMM_{20}, severe maternal morbidity measured without the blood product transfusion.

[a] Models adjusted for maternal age, race/ethnicity, education level, income level, year of delivery, and season of conception.

[b] The *P* value is for the interaction term between the extreme heat exposure variable and the effect modifier.

[c] This racial and ethnic group includes Native American Alaskan, Pacific Islander, other unspecified races/ethnicities, and multiple races/ethnicities.

[d] The group of smokers includes past smokers and smokers during pregnancy.

© 2023 Jiao A et al. *JAMA Network Open.*

eTable 4 The effect modification in associations between SMM_{20} and exposure to heat days during the entire pregnancy by green space exposure estimated using interaction terms.

	HD_P75 [a]		HD_P90 [a]		HD_P95 [a]	
	ORs (95% CIs)	*P* value [b]	ORs (95% CIs)	*P* value [b]	ORs (95% CIs)	*P* value [b]
Total green space exposure						
< 50th	1.168 (1.046-1.305)	0.36	1.166 (1.044-1.302)	0.99	1.223 (1.098-1.363)	0.45
≥ 50th	1.081 (0.954-1.225)		1.164 (1.035-1.310)		1.298 (1.159-1.454)	
Tree exposure						
< 50th	1.165 (1.048-1.295)	0.23	1.231 (1.109-1.367)	0.07	1.261 (1.137-1.399)	0.81
≥ 50th	1.050 (0.917-1.202)		1.057 (0.929-1.202)		1.237 (1.097-1.396)	
Low-lying vegetation exposure						
< 50th	1.113 (0.989-1.253)	0.70	1.112 (0.991-1.248)	0.24	1.202 (1.075-1.344)	0.23
≥ 50th	1.150 (1.025-1.290)		1.224 (1.094-1.369)		1.322 (1.185-1.475)	
Grass exposure						
< 50th	1.176 (1.022-1.353)	0.52	1.180 (1.032-1.349)	0.87	1.332 (1.175-1.509)	0.30
≥ 50th	1.111 (1.000-1.234)		1.164 (1.049-1.291)		1.224 (1.105-1.357)	

Abbreviations: CI, confidence interval; HD_P75, moderate heat days exceeding the 75th daily maximum temperature percentile; HD_P90, high heat days exceeding the 90th daily maximum temperature percentile; HD_P95, extreme heat days exceeding the 95th daily maximum temperature percentile; OR, odds ratio; SMM_{20}, severe maternal morbidity measured without the blood product transfusion.

[a] Models adjusted for maternal age, race/ethnicity, education level, income level, year of delivery, and season of conception.

[b] The *P* value is for the interaction term between the extreme heat exposure variable and the effect modifier.

© 2023 Jiao A et al. *JAMA Network Open.*

eTable 5 The estimated risks of SMM_{20} with 95% confidence intervals (CIs) associated with the high exposure to heat days during the entire pregnancy examined in the sensitivity analysis.

	HD_P75	HD_P90	HD_P95
Main model [a]	1.139 (1.048-1.237)	1.174 (1.083-1.273)	1.267 (1.171-1.370)
Unadjusted model			
Model 1 [b]	1.110 (1.023-1.204)	1.165 (1.077-1.262)	1.295 (1.199-1.399)
Model with more covariates			
Model 2 [c]	1.143 (1.051-1.244)	1.185 (1.091-1.287)	1.276 (1.178-1.383)
Model 3 [d]	1.141 (1.048-1.242)	1.190 (1.096-1.292)	1.258 (1.161-1.363)
Model 4 [e]	1.054 (0.961-1.155)	1.100 (1.006-1.203)	1.202 (1.103-1.310)
Model 5 [f]	1.054 (0.962-1.156)	1.101 (1.007-1.204)	1.203 (1.104-1.311)
Model accounting for spatial clustering			
Model 6 [g]	1.107 (1.003-1.223)	1.144 (1.039-1.259)	1.225 (1.114-1.346)
Model 7 [h]	1.128 (1.030-1.235)	1.163 (1.064-1.270)	1.236 (1.133-1.348)
Model accounting for multiple deliveries			
Model 8 [i]	1.152 (1.046-1.268)	1.187 (1.081-1.304)	1.269 (1.158-1.390)
Model with restricted gestational age			
Model 9 [j]	1.139 (1.048-1.237)	1.174 (1.083-1.273)	1.267 (1.171-1.370)
Model for daily maximum heat index			
Model 10 [k]	1.143 (1.050-1.245)	1.154 (1.063-1.252)	1.187 (1.094-1.288)
Cox proportional hazards model			
Model 11 [l]	1.192 (1.097-1.295)	1.226 (1.130-1.330)	1.288 (1.188-1.396)
Model after filling in missing residential data			
Model 12 [m]	1.137 (1.046-1.235)	1.165 (1.074-1.263)	1.262 (1.166-1.365)
Model using continuous exposure variable			
Model 13 [n]	1.124 (1.083-1.167)	1.370 (1.283-1.463)	1.545 (1.412-1.689)

Abbreviations: HD_P75, moderate heat days exceeding the 75th daily maximum temperature percentile; HD_P90, high heat days exceeding the 90th daily maximum temperature percentile; HD_P95, extreme heat days exceeding the 95th daily maximum temperature percentile; SMM_{20}, severe maternal morbidity measured without the blood product transfusion.

[a] Main model: model in the main analysis adjusted for maternal age, race/ethnicity, education level, income level, year of delivery, and season of conception.

[b] Model 1: model including only the exposure variable.

[c] Model 2: main model further adjusted for insurance type, pre-pregnancy body mass index, and parity.

[d] Model 3: main model further adjusted for insurance type, pre-pregnancy body mass index, parity, and preterm birth.

[e] Model 4: main model further adjusted for insurance type, pre-pregnancy body mass index, parity, preterm birth, and some pregnancy complications (i.e., gestational diabetes or gestational hypertension).

[f] Model 5: main model further adjusted for insurance type, pre-pregnancy body mass index, parity, preterm birth, some pregnancy complications (i.e., gestational diabetes or gestational hypertension), and some pre-existing medical conditions (i.e., pre-existing diabetes or chronic hypertension).

[g] Model 6: model with the county of residence as a random effect.

[h] Model 7: model with the zip code of residence as a random effect.

[i] Model 8: model including only the first delivery for each participant during the study period.

[j] Model 9: model excluding extreme post-term pregnancies > 43 weeks gestation (n of pregnancies = 61 in the total study population, 0.02%).

[k] Model 10: model with the daily maximum heat index applied as the alternative temperature metric to the daily maximum temperature for exposure assessment.

[l] Model 11: hazard ratios estimated based on Cox proportional hazards models with the gestational length in days as the temporal unit.

[m] Model 12: model for all subjects (n = 425,722) after filling in missing residential data.

[n] Model 13: the continuous exposure variable used in this model and results were represented by ORs of SMM_{20} per 10% increase in the proportion of heat days during the pregnancy.

© 2023 Jiao A et al. *JAMA Network Open.*

eTable 6 The estimated risks of SMM_{20} with 95% confidence intervals (CIs) associated with heatwaves under different definitions during the last gestational week in the sensitivity analysis.

	Main model [a]	Unadjusted model	Model with more covariates			
		Model 1 [b]	Model 2 [c]	Model 3 [d]	Model 4 [e]	Model 5 [f]
HWD1 (DMT > 75th and ≥ 2 days)	1.317 (1.174-1.478)	1.284 (1.145-1.440)	1.312 (1.167-1.475)	1.237 (1.101-1.391)	1.196 (1.057-1.353)	1.196 (1.057-1.353)
HWD2 (DMT > 75th and ≥ 3 days)	1.413 (1.244-1.605)	1.371 (1.208-1.557)	1.415 (1.244-1.610)	1.340 (1.178-1.525)	1.307 (1.140-1.498)	1.306 (1.139-1.497)
HWD3 (DMT > 75th and ≥ 4 days)	1.453 (1.253-1.685)	1.410 (1.216-1.634)	1.452 (1.250-1.688)	1.379 (1.187-1.603)	1.345 (1.146-1.578)	1.345 (1.146-1.578)
HWD4 (DMT > 90th and ≥ 2 days)	1.444 (1.222-1.706)	1.443 (1.222-1.704)	1.449 (1.224-1.716)	1.386 (1.170-1.641)	1.303 (1.085-1.565)	1.303 (1.085-1.564)
HWD5 (DMT > 90th and ≥ 3 days)	1.441 (1.153-1.800)	1.405 (1.125-1.754)	1.433 (1.142-1.798)	1.375 (1.095-1.726)	1.268 (0.986-1.630)	1.269 (0.987-1.632)
HWD6 (DMT > 90th and ≥ 4 days)	1.482 (1.110-1.980)	1.446 (1.084-1.929)	1.468 (1.092-1.972)	1.395 (1.037-1.877)	1.205 (0.855-1.699)	1.201 (0.852-1.693)
HWD7 (DMT > 95th and ≥ 2 days)	1.365 (1.070-1.741)	1.419 (1.114-1.807)	1.368 (1.069-1.751)	1.318 (1.029-1.688)	1.134 (0.855-1.503)	1.133 (0.854-1.502)
HWD8 (DMT > 95th and ≥ 3 days)	1.758 (1.266-2.441)	1.726 (1.244-2.394)	1.771 (1.270-2.471)	1.712 (1.226-2.389)	1.427 (0.958-2.126)	1.431 (0.961-2.131)
HWD9 (DMT > 95th and ≥ 4 days)	2.391 (1.618-3.535)	2.347 (1.590-3.463)	2.378 (1.596-3.542)	2.312 (1.550-3.447)	1.991 (1.229-3.227)	1.995 (1.231-3.234)

Abbreviations: DMT, daily maximum temperature; HWD, heatwave definition; SMM_{20}, severe maternal morbidity measured without the blood product transfusion.
[a] Main model: model in the main analysis adjusting for maternal age, race/ethnicity, education level, income level, and year of delivery.
[b] Model 1: model including only the exposure variable.
[c] Model 2: main model further adjusted for insurance type, pre-pregnancy body mass index, and parity.
[d] Model 3: main model further adjusted for insurance type, pre-pregnancy body mass index, parity, and preterm birth.
[e] Model 4: main model further adjusted for insurance type, pre-pregnancy body mass index, parity, preterm birth, and some pregnancy complications (i.e., gestational diabetes or gestational hypertension).
[f] Model 5: main model further adjusted for insurance type, pre-pregnancy body mass index, parity, preterm birth, some pregnancy complications (i.e., gestational diabetes or gestational hypertension), and some pre-existing medical conditions (i.e., pre-existing diabetes or chronic hypertension).
[g] Model 6: model with the county of residence as a random effect.
[h] Model 7: model with the zip code of residence as a random effect.
[I] Model 8: model including only the first delivery for each participant during the study period.
[j] Model 9: model excluding extreme post-term pregnancies > 43 weeks gestation (n of pregnancies = 61 in the total study population, 0.02%).
[k] Model 10: model with the daily maximum heat index applied as the alternative temperature metric to the daily maximum temperature for exposure assessment.
[l] Model 11: hazard ratios estimated based on Cox proportional hazards models with the gestational length in days as the temporal unit.
[m] Model 12: model for all subjects (n = 425,722) after filling in missing residential data.

© 2023 Jiao A et al. *JAMA Network Open.*

eTable 6 The estimated risks of SMM_{20} with 95% confidence intervals (CIs) associated with heatwaves under different definitions during the last gestational week in the sensitivity analysis (continued).

	Main model [a]	Model accounting for spatial clustering		Model accounting for multiple deliveries	Model with restricted gestational age
		Model 6 [g]	Model 7 [h]	Model 8 [i]	Model 9 [j]
HWD1 (DMT > 75th and ≥ 2 days)	1.317 (1.174-1.478)	1.120 (0.994-1.263)	1.122 (0.999-1.260)	1.314 (1.148-1.505)	1.316 (1.173-1.477)
HWD2 (DMT > 75th and ≥ 3 days)	1.413 (1.244-1.605)	1.221 (1.070-1.393)	1.219 (1.073-1.386)	1.406 (1.210-1.634)	1.414 (1.245-1.606)
HWD3 (DMT > 75th and ≥ 4 days)	1.453 (1.253-1.685)	1.271 (1.091-1.480)	1.267 (1.091-1.471)	1.507 (1.269-1.790)	1.455 (1.254-1.687)
HWD4 (DMT > 90th and ≥ 2 days)	1.444 (1.222-1.706)	1.281 (1.081-1.517)	1.278 (1.081-1.511)	1.396 (1.142-1.706)	1.446 (1.224-1.709)
HWD5 (DMT > 90th and ≥ 3 days)	1.441 (1.153-1.800)	1.263 (1.008-1.583)	1.258 (1.005-1.575)	1.541 (1.193-1.990)	1.446 (1.157-1.806)
HWD6 (DMT > 90th and ≥ 4 days)	1.482 (1.110-1.980)	1.295 (0.966-1.735)	1.277 (0.952-1.714)	1.562 (1.118-2.182)	1.490 (1.116-1.990)
HWD7 (DMT > 95th and ≥ 2 days)	1.365 (1.070-1.741)	1.218 (0.955-1.553)	1.212 (0.950-1.545)	1.443 (1.087-1.915)	1.367 (1.072-1.744)
HWD8 (DMT > 95th and ≥ 3 days)	1.758 (1.266-2.441)	1.494 (1.072-2.082)	1.473 (1.054-2.059)	1.945 (1.342-2.820)	1.760 (1.268-2.445)
HWD9 (DMT > 95th and ≥ 4 days)	2.391 (1.618-3.535)	2.027 (1.367-3.005)	1.985 (1.330-2.964)	2.625 (1.680-4.101)	2.389 (1.616-3.531)

Abbreviations: DMT, daily maximum temperature; HWD, heatwave definition; SMM_{20}, severe maternal morbidity measured without the blood product transfusion.
[a] Main model: model in the main analysis adjusting for maternal age, race/ethnicity, education level, income level, and year of delivery.
[b] Model 1: model including only the exposure variable.
[c] Model 2: main model further adjusted for insurance type, pre-pregnancy body mass index, and parity.
[d] Model 3: main model further adjusted for insurance type, pre-pregnancy body mass index, parity, and preterm birth.
[e] Model 4: main model further adjusted for insurance type, pre-pregnancy body mass index, parity, preterm birth, and some pregnancy complications (i.e., gestational diabetes or gestational hypertension).
[f] Model 5: main model further adjusted for insurance type, pre-pregnancy body mass index, parity, preterm birth, some pregnancy complications (i.e., gestational diabetes or gestational hypertension), and some pre-existing medical conditions (i.e., pre-existing diabetes or chronic hypertension).
[g] Model 6: model with the county of residence as a random effect.
[h] Model 7: model with the zip code of residence as a random effect.
[I] Model 8: model including only the first delivery for each participant during the study period.
[j] Model 9: model excluding extreme post-term pregnancies > 43 weeks gestation (n of pregnancies = 61 in the total study population, 0.02%).
[k] Model 10: model with the daily maximum heat index applied as the alternative temperature metric to the daily maximum temperature for exposure assessment.
[l] Model 11: hazard ratios estimated based on Cox proportional hazards models with the gestational length in days as the temporal unit.
[m] Model 12: model for all subjects (n = 425,722) after filling in missing residential data.

© 2023 Jiao A et al. *JAMA Network Open.*

eTable 6 The estimated risks of SMM_{20} with 95% confidence intervals (CIs) associated with heatwaves under different definitions during the last gestational week in the sensitivity analysis (continued).

	Main model [a]	Model for daily maximum heat index	Cox proportional hazards model	Model after filling in missing residential data
		Model 10 [k]	Model 11 [l]	Model 12 [m]
HWD1 (DMT > 75th and ≥ 2 days)	1.317 (1.174-1.478)	1.157 (1.034-1.294)	1.146 (1.024-1.282)	1.331 (1.190-1.489)
HWD2 (DMT > 75th and ≥ 3 days)	1.413 (1.244-1.605)	1.187 (1.048-1.345)	1.244 (1.098-1.410)	1.412 (1.247-1.599)
HWD3 (DMT > 75th and ≥ 4 days)	1.453 (1.253-1.685)	1.179 (1.020-1.364)	1.300 (1.123-1.505)	1.427 (1.233-1.651)
HWD4 (DMT > 90th and ≥ 2 days)	1.444 (1.222-1.706)	1.273 (1.088-1.489)	1.320 (1.120-1.557)	1.463 (1.244-1.722)
HWD5 (DMT > 90th and ≥ 3 days)	1.441 (1.153-1.800)	1.279 (1.046-1.562)	1.323 (1.061-1.649)	1.495 (1.207-1.852)
HWD6 (DMT > 90th and ≥ 4 days)	1.482 (1.110-1.980)	1.351 (1.046-1.745)	1.368 (1.025-1.825)	1.534 (1.161-2.026)
HWD7 (DMT > 95th and ≥ 2 days)	1.365 (1.070-1.741)	1.518 (1.192-1.933)	1.276 (1.004-1.621)	1.418 (1.123-1.792)
HWD8 (DMT > 95th and ≥ 3 days)	1.758 (1.266-2.441)	1.268 (0.871-1.848)	1.585 (1.142-2.198)	1.808 (1.318-2.479)
HWD9 (DMT > 95th and ≥ 4 days)	2.391 (1.618-3.535)	0.754 (0.376-1.515)	2.171 (1.471-3.204)	2.441 (1.674-3.558)

Abbreviations: DMT, daily maximum temperature; HWD, heatwave definition; SMM_{20}, severe maternal morbidity measured without the blood product transfusion.
[a] Main model: model in the main analysis adjusting for maternal age, race/ethnicity, education level, income level, and year of delivery.
[b] Model 1: model including only the exposure variable.
[c] Model 2: main model further adjusted for insurance type, pre-pregnancy body mass index, and parity.
[d] Model 3: main model further adjusted for insurance type, pre-pregnancy body mass index, parity, and preterm birth.
[e] Model 4: main model further adjusted for insurance type, pre-pregnancy body mass index, parity, preterm birth, and some pregnancy complications (i.e., gestational diabetes or gestational hypertension).
[f] Model 5: main model further adjusted for insurance type, pre-pregnancy body mass index, parity, preterm birth, some pregnancy complications (i.e., gestational diabetes or gestational hypertension), and some pre-existing medical conditions (i.e., pre-existing diabetes or chronic hypertension).
[g] Model 6: model with the county of residence as a random effect.
[h] Model 7: model with the zip code of residence as a random effect.
[I] Model 8: model including only the first delivery for each participant during the study period.
[j] Model 9: model excluding extreme post-term pregnancies > 43 weeks gestation (n of pregnancies = 61 in the total study population, 0.02%).
[k] Model 10: model with the daily maximum heat index applied as the alternative temperature metric to the daily maximum temperature for exposure assessment.
[l] Model 11: hazard ratios estimated based on Cox proportional hazards models with the gestational length in days as the temporal unit.
[m] Model 12: model for all subjects (n = 425,722) after filling in missing residential data.

© 2023 Jiao A et al. *JAMA Network Open.*

eTable 7 The adjusted odds ratios (ORs) with 95% confidence intervals (CIs) of other SMM outcomes associated with the high exposure to heat days (≥ 80th) during the entire pregnancy.

	SMM_{21} [a]	SMM_{blood_transf} [a]	SMM_{DIC} [a]	SMM_{sepsis} [a]
HD_P75	0.996 (0.939-1.058)	0.870 (0.798-0.948)	0.907 (0.794-1.038)	1.313 (1.072-1.607)
HD_P90	1.001 (0.944-1.062)	0.850 (0.780-0.925)	0.923 (0.810-1.052)	1.279 (1.050-1.559)
HD_P95	1.057 (0.998-1.119)	0.874 (0.804-0.951)	1.040 (0.918-1.179)	1.371 (1.134-1.658)

Abbreviations: HD_P75, moderate heat days exceeding the 75th daily maximum temperature percentile; HD_P90, high heat days exceeding the 90th daily maximum temperature percentile; HD_P95, extreme heat days exceeding the 95th daily maximum temperature percentile; SMM_{21}, severe maternal morbidity measured with all 21 indicators; SMM_{blood_transf}, severe maternal morbidity cases with the blood product transfusion as the only indicator; SMM_{DIC}, the disseminated intervascular coagulation in severe maternal morbidity; SMM_{sepsis}, the sepsis in severe maternal morbidity.
[a] Models adjusted for maternal age, race/ethnicity, education level, income level, year of delivery, and season of conception.

© 2023 Jiao A et al. *JAMA Network Open.*

eTable 8 The adjusted odds ratios (ORs) with 95% confidence intervals (CIs) of other SMM outcomes associated with heatwaves under different definitions during the last gestational week.

	SMM_{21} [a]	SMM_{blood_transf} [a]	SMM_{DIC} [a]	SMM_{sepsis} [a]
HWD1 (DMT > 75th and ≥ 2 days)	1.141 (1.048-1.242)	0.969 (0.854-1.099)	1.009 (0.842-1.210)	1.508 (1.131-2.010)
HWD2 (DMT > 75th and ≥ 3 days)	1.179 (1.071-1.298)	0.950 (0.821-1.101)	1.204 (0.988-1.467)	1.483 (1.074-2.049)
HWD3 (DMT > 75th and ≥ 4 days)	1.183 (1.056-1.326)	0.916 (0.765-1.096)	1.178 (0.929-1.494)	1.880 (1.328-2.660)
HWD4 (DMT > 90th and ≥ 2 days)	1.152 (1.011-1.314)	0.855 (0.691-1.057)	1.109 (0.843-1.460)	1.959 (1.345-2.854)
HWD5 (DMT > 90th and ≥ 3 days)	1.155 (0.969-1.377)	0.859 (0.645-1.144)	1.224 (0.857-1.749)	2.575 (1.653-4.009)
HWD6 (DMT > 90th and ≥ 4 days)	1.240 (0.990-1.553)	0.984 (0.688-1.407)	1.024 (0.613-1.712)	3.061 (1.804-5.193)
HWD7 (DMT > 95th and ≥ 2 days)	1.185 (0.982-1.430)	0.989 (0.736-1.329)	0.949 (0.620-1.455)	2.142 (1.318-3.479)
HWD8 (DMT > 95th and ≥ 3 days)	1.426 (1.100-1.850)	1.082 (0.709-1.652)	1.051 (0.562-1.965)	3.612 (2.009-6.495)
HWD9 (DMT > 95th and ≥ 4 days)	1.833 (1.333-2.522)	1.260 (0.728-2.181)	1.235 (0.551-2.767)	4.574 (2.241-9.334)

Abbreviations: DMT, daily maximum temperature; HWD, heatwave definition; SMM_{21}, severe maternal morbidity measured with all 21 indicators; SMM_{blood_transf}, severe maternal morbidity cases with the blood product transfusion as the only indicator; SMM_{DIC}, the disseminated intervascular coagulation in severe maternal morbidity; SMM_{sepsis}, the sepsis in severe maternal morbidity.

[a] Models adjusted for maternal age, race/ethnicity, education level, income level, and year of delivery.

© 2023 Jiao A et al. *JAMA Network Open.*

Reference

1. Jiao A, Sun Y, Avila C, et al. Maternal exposure to ambient air pollution mixture and premature rupture of membranes: Evidence from a large cohort in Southern California (2008-2018). *Environ Int*. Jul 2023;177:108030. doi:10.1016/j.envint.2023.108030
2. Sun Y, Li X, Benmarhnia T, et al. Exposure to air pollutant mixture and gestational diabetes mellitus in Southern California: Results from electronic health record data of a large pregnancy cohort. *Environ Int*. Sep 24 2021;158:106888. doi:10.1016/j.envint.2021.106888
3. Sun Y, Molitor J, Benmarhnia T, et al. Association between urban green space and postpartum depression, and the role of physical activity: a retrospective cohort study in Southern California. *The Lancet Regional Health - Americas*. 2023:100462. doi:10.1016/j.lana.2023.100462
4. Callaghan WM, Mackay AP, Berg CJ. Identification of severe maternal morbidity during delivery hospitalizations, United States, 1991-2003. *Am J Obstet Gynecol*. Aug 2008;199(2):133 e1-8. doi:10.1016/j.ajog.2007.12.020
5. Creanga AA, Bateman BT, Kuklina EV, Callaghan WM. Racial and ethnic disparities in severe maternal morbidity: a multistate analysis, 2008-2010. *Am J Obstet Gynecol*. May 2014;210(5):435 e1-8. doi:10.1016/j.ajog.2013.11.039
6. Grobman WA, Bailit JL, Rice MM, et al. Frequency of and factors associated with severe maternal morbidity. *Obstet Gynecol*. Apr 2014;123(4):804-10. doi:10.1097/AOG.0000000000000173
7. Kilpatrick SJ, Abreo A, Gould J, Greene N, Main EK. Confirmed severe maternal morbidity is associated with high rate of preterm delivery. *Am J Obstet Gynecol*. Aug 2016;215(2):233 e1-7. doi:10.1016/j.ajog.2016.02.026
8. Gibson C, Rohan AM, Gillespie KH. Severe Maternal Morbidity During Delivery Hospitalizations. *WMJ*. Dec 2017;116(5):215-220.
9. Admon LK, Winkelman TNA, Zivin K, Terplan M, Mhyre JM, Dalton VK. Racial and Ethnic Disparities in the Incidence of Severe Maternal Morbidity in the United States, 2012-2015. *Obstet Gynecol*. Nov 2018;132(5):1158-1166. doi:10.1097/AOG.0000000000002937
10. Hitti J, Sienas L, Walker S, Benedetti TJ, Easterling T. Contribution of hypertension to severe maternal morbidity. *Am J Obstet Gynecol*. Oct 2018;219(4):405 e1-405 e7. doi:10.1016/j.ajog.2018.07.002
11. Howell EA. Reducing Disparities in Severe Maternal Morbidity and Mortality. *Clinical obstetrics and gynecology*. Jun 2018;61(2):387-399. doi:10.1097/GRF.0000000000000349
12. Shamshirsaz AA, Dildy GA. Reducing Maternal Mortality and Severe Maternal Morbidity: The Role of Critical Care. *Clinical obstetrics and gynecology*. Jun 2018;61(2):359-371. doi:10.1097/GRF.0000000000000370
13. Conrey EJ, Manning SE, Shellhaas C, et al. Severe Maternal Morbidity, A Tale of 2 States Using Data for Action-Ohio and Massachusetts. *Matern Child Health J*. Aug 2019;23(8):989-995. doi:10.1007/s10995-019-02744-1
14. Guglielminotti J, Landau R, Wong CA, Li G. Patient-, Hospital-, and Neighborhood-Level Factors Associated with Severe Maternal Morbidity During Childbirth: A Cross-Sectional Study in New York State 2013-2014. *Matern Child Health J*. Jan 2019;23(1):82-91. doi:10.1007/s10995-018-2596-9
15. Leonard SA, Main EK, Scott KA, Profit J, Carmichael SL. Racial and ethnic disparities in severe maternal morbidity prevalence and trends. *Ann Epidemiol*. May 2019;33:30-36. doi:10.1016/j.annepidem.2019.02.007
16. Liese KL, Mogos M, Abboud S, Decocker K, Koch AR, Geller SE. Racial and Ethnic Disparities in Severe Maternal Morbidity in the United States. *J Racial Ethn Health Disparities*. Aug 2019;6(4):790-798. doi:10.1007/s40615-019-00577-w
17. Brown CC, Adams CE, George KE, Moore JE. Associations Between Comorbidities and Severe Maternal Morbidity. *Obstet Gynecol*. Nov 2020;136(5):892-901. doi:10.1097/AOG.0000000000004057
18. Howell EA, Egorova NN, Janevic T, et al. Race and Ethnicity, Medical Insurance, and Within-Hospital Severe Maternal Morbidity Disparities. *Obstet Gynecol*. Feb 2020;135(2):285-293. doi:10.1097/AOG.0000000000003667
19. Chen J, Cox S, Kuklina EV, Ferre C, Barfield W, Li R. Assessment of Incidence and Factors Associated With Severe Maternal Morbidity After Delivery Discharge Among Women in the US. *JAMA network open*. Feb 1 2021;4(2):e2036148. doi:10.1001/jamanetworkopen.2020.36148
20. Frey HA, Ashmead R, Farmer A, et al. Association of Prepregnancy Body Mass Index With Risk of Severe Maternal Morbidity and Mortality Among Medicaid Beneficiaries. *JAMA network open*. Jun 1 2022;5(6):e2218986. doi:10.1001/jamanetworkopen.2022.18986
21. Muchomba FM, Teitler J, Reichman NE. Association Between Housing Affordability and Severe Maternal Morbidity. *JAMA network open*. Nov 1 2022;5(11):e2243225. doi:10.1001/jamanetworkopen.2022.43225

© 2023 Jiao A et al. *JAMA Network Open.*

22. Oakley LP, Li X, Tartof SY, Wilkes-Grundy M, Fassett MJ, Lawrence JM. Racial Disparities in Severe Maternal Morbidity in an Integrated Health Care System, Southern California, 2008-2017. *Womens Health Issues*. Feb 3 2023;doi:10.1016/j.whi.2023.01.001

23. Howland RE, Angley M, Won SH, et al. Determinants of Severe Maternal Morbidity and Its Racial/Ethnic Disparities in New York City, 2008-2012. *Matern Child Health J*. Mar 2019;23(3):346-355. doi:10.1007/s10995-018-2682-z

24. Harden SR, Runkle JD, Sugg MM. An Exploratory Spatiotemporal Analysis of Socio-Environmental Patterns in Severe Maternal Morbidity. *Matern Child Health J*. May 2022;26(5):1077-1086. doi:10.1007/s10995-021-03330-0

25. Jiao A, Sun Y, Sacks DA, et al. The Role of Extreme Heat Exposure on Premature Rupture of Membranes in Southern California: A study from a Large Pregnancy Cohort. *Environment International*. 2023/02/13/ 2023:107824. doi:https://doi.org/10.1016/j.envint.2023.107824

© 2023 Jiao A et al. *JAMA Network Open.*
