# Supplementary material for: Analysis of Heat Exposure During Pregnancy and Severe Maternal Morbidity
Source: JAMA Netw Open. 2023 Sep 7;6(9):e2332780. doi: 10.1001/jamanetworkopen.2023.32780 (PMC10485728; doi:10.1001/jamanetworkopen.2023.32780)
Supplement: Supplement 2. — Data Sharing Statement [file jamanetwopen-e2332780-s002.pdf]

Data Sharing Statement

Jiao. Analysis of Heat Exposure During Pregnancy and Severe Maternal Morbidity. *JAMA Netw Open*. Published September 07, 2023. doi:10.1001/jamanetworkopen.2023.32780

Data
Data available: No

Additional Information
Explanation for why data not available: We use individual-level electronic health record data from a healthcare system; these data are confidential and protected by specific Institutional Review Board protocols.
